# Friction-induced nanofabrication method to produce protrusive nanostructures on quartz

**DOI:** 10.1186/1556-276X-6-310

**Published:** 2011-04-07

**Authors:** Chenfei Song, Xiaoying Li, Bingjun Yu, Hanshan Dong, Linmao Qian, Zhongrong Zhou

**Affiliations:** 1 Tribology Research Institute, National Traction Power Laboratory, Southwest Jiaotong University, Chengdu, Sichuan Province 610031, People's Republic of China; 2 School of Metallurgy and Materials, The University of Birmingham, Birmingham B15 2TT, UK

## Abstract

In this paper, a new friction-induced nanofabrication method is presented to fabricate protrusive nanostructures on quartz surfaces through scratching a diamond tip under given normal loads. The nanostructures, such as nanodots, nanolines, surface mesas and nanowords, can be produced on the target surface by programming the tip traces according to the demanded patterns. The height of these nanostructures increases with the increase of the number of scratching cycles or the normal load. Transmission electron microscope observations indicated that the lattice distortion and dislocations induced by the mechanical interaction may have played a dominating role in the formation of the protrusive nanostructures on quartz surfaces. Further analysis reveals that during scratching, a contact pressure ranged from 0.4*P*_y _to *P*_y _(*P*_y _is the critical yield pressure of quartz) is apt to produce protuberant nanostructures on quartz under the given experimental conditions. Finally, it is of great interest to find that the protrusive nanostructures can be selectively dissolved in 20% KOH solution. Since the nanowords can be easily 'written' by friction-induced fabrication and 'erased' through selective etching on a quartz surface, this friction-induced method opens up new opportunities for future nanofabrication.

## Introduction

Due to its high fundamental frequencies, high Q property and excellent piezoelectric behaviour, quartz has been widely used to produce sensors in micro/nano electromechanical systems (MEMS/NEMS), such as accelerometer, pressure sensor, gyro sensor, crystal oscillator and tuning fork biosensor, etc. [1-5]. Moreover, quartz is also an ideal substrate material for MEMS/NEMS because of its well-known electrical insulation and chemical stability [6]. As the scale-down of device dimensions continues, it is essential to explore new nanofabrication methods, especially for nanoscale quartz devices.

General methods for the nanofabrication of quartz include chemical etching, plasma etching and reactive ion etching [7-9]. However, the use of these techniques tends to be limited by such disadvantages as environmental pollution, poor resolution and low efficiency [10]. Focused ion beam (FIB) technology is advantageous for material processing at nanoscale because of its high machining precision [11], but the ion beam may cause undesirable materials degradation and the equipment is costly [12,13]. In recent years, atomic force microscope (AFM) and scanning tunnelling microscope (STM) have been utilized for nanofabrication [14]. The AFM tips are used as sharp tools to cut and remove materials, thus forming nano-grooves on the target surface [15]. However, it is difficult, if not impossible, to fabricate protrusive nanostructures using AFM. Combining anodic oxidation with STM can be used to process protuberant structures on the surfaces of conductors and semiconductors under a given voltage [16,17]. Nevertheless, the anodic oxidation process is invalid for nonconductive STM tips or for insulator surfaces. Therefore, by virtue of the high precision and multifunction of AFM, it is of great importance to develop an accurate and straightforward method for fabricating protuberant nanostructures on insulator surfaces based on the proximal probe method.

In the present study, a new friction-induced nanofabrication method to produce protrusive nanostructures on a quartz surface has been developed by directly scratching a diamond tip on the quartz surface under a given normal load. The capability of this nanofabrication method has been demonstrated by various nanostructures including nanodots, nanolines, isolated mesas, nanowords and so on. The effect of the applied normal load and the number of scratching cycles on the height of nanolines was studied. The generation mechanism of the protuberant nanostructures was discussed and the chemical activity of nanolines on quartz was investigated. Clearly, the friction-induced nanofabrication method may shed new light on nanotechnology.

## Material and experimental details

### Material

The monocrystalline quartz wafers (AT-cut) with a thickness of 0.5 mm were purchased from Semiconductor Wafer, Inc. (Hsinchu, Taiwan). By using an atomic force microscope (AFM, SPI3800N, Seiko, Tokyo, Japan), the root-mean-square (RMS) roughness of the quartz wafers was measured to be 0.25 nm over a 2 μm × 2 μm area. The chemical binding and crystal state of the quartz material were detected by an X-ray photoelectron spectroscope (XPS, XSAM 800, CRATOS CO., Manchester, UK) and an X-ray diffraction machine (XRD, Philips X'Pert PRO, PANalytical, Almelo, Netherlands), respectively. The results indicate that the monocrystalline quartz consists of pure SiO_2 _and its crystal plane is  Before the fabrication, the quartz samples were ultrasonically cleaned in methanol, ethanol and deionized water for 10 min. To etch the quartz sample, a KOH solution (20% in weight) was prepared using solid KOH (analytical purity) and deionized water.

### Fabrication methods

All the fabrications were performed by an AFM equipped with a vacuum chamber. If not specially mentioned, a pyramidal diamond tip (Micro Star Technologies, Huntsville, USA) with curvature radius *R *of 300 nm was used for fabrication. The spring constant of the cantilever was calibrated as 203 N/m [18]. After the fabrication, the topography of protrusive nanostructures was scanned in situ by a Si_3_N_4 _tip with a spring constant of 0.1 N/m (MLCT, Veeco, Plainview, USA). Two scratching modes were adopted in this study: bidirectional line-scratch for producing linear protrusive nanostructures and scanning-scratch for area protrusive nanostructures (surface mesas) [19-21]. The length of nanolines was controlled by the displacement amplitude *D *of the diamond tip on samples. If *D *shortens to several tens of nanometers under line-scratch, a nanodot would be made. In both modes, the scratching frequency was set at 2 Hz, the temperature was controlled at 20 ± 3°C and the relative humidity was ranged between 50 ± 5%.

To investigate the effect of the number of line-scratch cycles *N *on the generation of protrusive nanostructures, the scratching tests were performed on quartz surface under an applied normal load *F*_n _= 5 μN and various *N *between 10 and 150. Moreover, the effect of *F*_n _on the generation of protrusive nanostructures was studied by 100 line-scratch cycles on quartz surface under various *F*_n _of 3 to 26 μN. During the tests, the displacement amplitude of the line-scratch was 4 μm and the sliding speed was 32 μm/s if not specifically mentioned. Nanodots were produced by line-scratching with *F*_n _= 6 μN, *N *= 100 and *D *= 80 nm. The surface mesa with size of 3 μm × 3 μm was fabricated under *F*_n _= 6 μN and *N *= 4. By using a dull diamond tip with curvature radius of 500 nm, the letters 'NANO' were written at *F*_n _= 30 μN, *N *= 100. To understand the generation mechanism of the protrusive nanostructures, some nanolines were created on quartz surface in vacuum with a pressure better than 2.7 × 10^-4 ^Pa.

### TEM characterization

Cross-sectional transmission electron microscope (XTEM) sample was prepared by Quanta 3D FEG FIB miller (FEI Company, Hillsboro, USA). Prior to milling, a protective platinum coating was deposited by 5 kV electron-beam firstly and then 30 kV at 0.5 nA ion-beam on the top of nanolines. Milling was performed with low beam currents from 15 to 5 nA at 30 kV and final thinning at 1 to 0.1 nA, 30 kV, finished with a final polish mills at 5 kV 48 pA. A JEOL JEM-2100 LaB6 TEM (JEOL Ltd., Tokyo, Japan) with the operating voltage of 80 kV was used to characterise the cross-sectional protrusive nanostructures.

## Experimental results

### Fabrication of nanolines on quartz surface

When scratching a surface using a sharp diamond tip, the generation of grooves was usually observed in the scratched area [15]. However, protrusive nanostructures have been generated in the present study under the given conditions. Since the formation of the protrusive nanostructures on quartz surface has not been observed during indentation tests, the sliding and friction seem to be the necessary conditions for the generation of protrusive nanostructures. Therefore, the process can be viewed as friction-induced nanofabrication.

**Figure 1:**
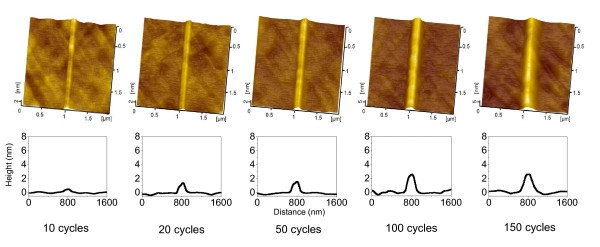
**Nanolines produced by various number of scratching cycles**. AFM images (top) and cross-sectional profiles (bottom) of the friction-induced nanolines on quartz surface created after various number of scratching cycles. The applied normal load *F*_n _is 5 μN.

**Figure 2:**
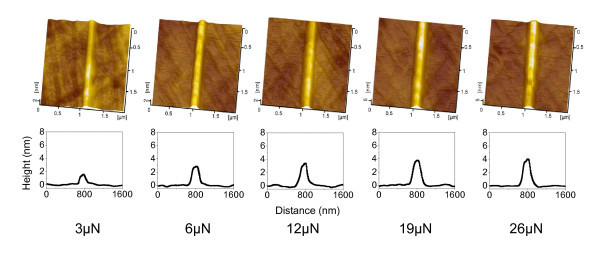
**Nanolines produced under various normal loads**. AFM images (top) and cross-sectional profiles (bottom) of the friction-induced nanolines on quartz surface created by scratching under various normal loads. The number of scratching cycles *N *= 100.

It was observed that both the number of scratching cycles *N *and the normal load *F*_n _contributed greatly to the creation of nanolines. As shown in Figure 1, the AFM images revealed that after 10 cycles of line-scratch test on quartz under a normal load *F*_n _of 5 μN in atmosphere, a nanoline was generated along the scratching trace. With the increase in the number of line-scratch cycles *N *from 10 to 150, the height of the nanolines was found to grow from 0.6 to 2.8 nm. After 100 repeated line-scratch tests, the height of the nanolines in Figure 2 increased from 1.6 to 4.0 nm with the increase in *F*_n _from 3 to 26 μN.

**Figure 3:**
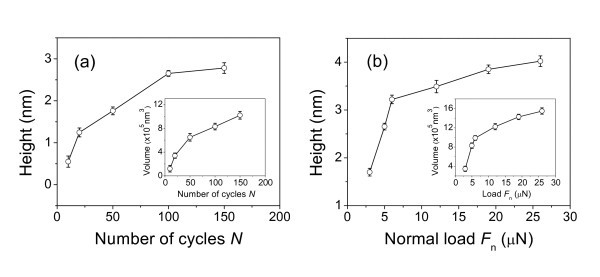
**Effect of the number of scratching cycles and normal load on the nanofabrication**. The effect of **(a) **the number of scratching cycles *N *and **(b) **normal load *F*_n _on the height and volume of the friction-induced nanolines on quartz surface.

To investigate the effect of line-scratching cycles *N *and normal load *F*_n _on the fabrication, both the height and volume of the nanolines were calculated with the original quartz surface as the base level. As shown in Figure 3a, under a normal load of 5 μN, the height of nanolines increases quickly in the initial scratching cycles and attains 2.8 nm after 150 line-scratch cycles. After 100 line-scratch cycles, the height of nanolines increases rapidly under low loads then reaches 4.0 nm under *F*_n_= 26 μN (Figure 3b).

### Fabrication of various nanostructures on quartz surface

**Figure 4:**
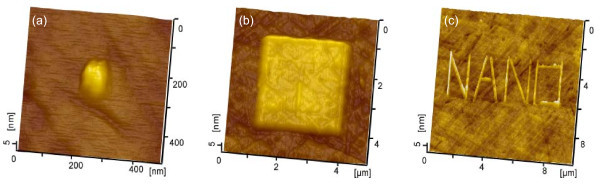
**Fabrication of various nanostructures on quartz surface**. **(a) **nanodot created by line-scratch at *F*_n _= 6 μN, *N *= 100 and *D *= 80 nm, **(b) **surface mesa generated by scanning-scratch at *F*_n _= 6 μN and *N *= 4 and **(c) **nanoletters produced by a dull diamond tip with *R *= 500 nm at *F*_n _= 30 μN and *N *= 100.

The friction-induced method can easily fabricate various nanostructures on quartz surfaces. For example, nanodots can be produced by line-scratching with short scratch displacement amplitude *D*. As shown in Figure 4a, the nanodot with a height of 2.5 nm and a diameter of 200 nm was fabricated by line-scratching at *D *= 80 nm. Surface isolated mesas can be created by scanning-scratch. In Figure 4b, when the scan area was set as 3 μm × 3 μm, a surface mesa with a height of 2.9 nm was made on a quartz surface under *F*_n _= 6 μN and *N *= 4. Surface patterned structures can also be fabricated by programming the tip trace in terms of the demanded pattern. In Figure 4c, the word 'NANO' on a quartz surface was written by the friction-induced fabrication process through connecting the nanolines together. The height of the letter is 1.5 nm and the width is about 300 nm.

## Discussion

### Generation mechanism of the friction-induced nanostructures on quartz

It was reported that the protrusive nanostructures could also be produced by scratching on silicon surface [19,20,22]. Andoh and coworkers [22] suggested that the formation of silicon hillocks was mainly attributed to the chemical reactions in the atmosphere. However, our recent research indicated that the friction-induced silicon hillock can be fabricated in vacuum with a pressure better than 2.7 × 10^-4 ^Pa [19]. Based on the TEM observation on the cross-sectional microstructure of the silicon nanolines, mechanical interaction between the tip and the materials surfaces has been proved to play a dominant role in the generation of silicon hillocks [19,20].

**Figure 5:**
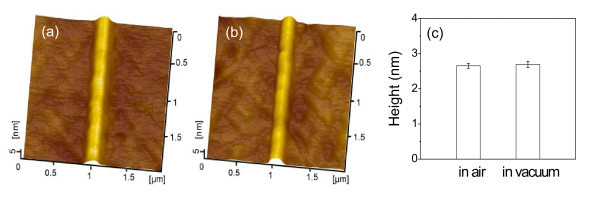
**Nanofabrication in air and in vacuum**. AFM images of nanolines on quartz surface created **(a) **in air and **(b) **in vacuum, **(c) **comparison of the height of nanolines on quartz fabricated in air and in vacuum.

In order to elucidate the effect of the oxidation and the oxygen atmosphere on the formation of hillock-like nanostructures on quartz surface, nanolines have been fabricated both in air and in vacuum with a pressure lower than 2.7 × 10^-4 ^Pa. As shown in Figure 5, after 100 line-scratch cycles under *F*_n _= 5 μN, the height of the nanolines created in vacuum is almost the same as those created in air. Clearly, the oxygen atmosphere shows a neglectable effect on the friction-induced nanofabrication on quartz surface. Similar to the formation of hillocks on silicon surfaces, the mechanical interaction may be the essential factor for the generation of protrusive nanostructures on quartz surfaces.

**Figure 6:**
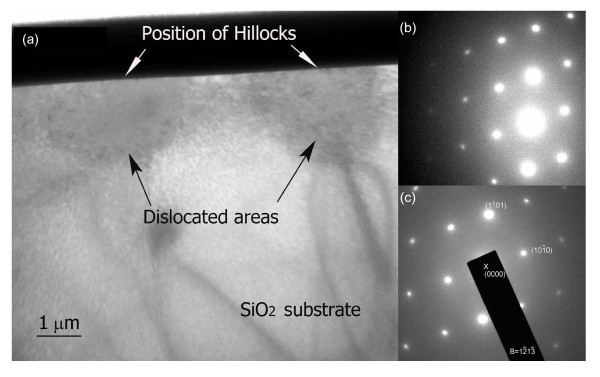
**XTEM and SAED observation of nanolines on quartz**. **(a) **XTEM image showing the cross-sectional structure of two typical nanolines formed on quartz surface, **(b) **the SAED pattern from the original area and **(c) **the SAED pattern from the deformed area.

To further understand the mechanism of the formation of the protrusive nanostructures, the microstructure of nanolines of about 4 nm in height was analysed by the cross-sectional transmission electron microscope (XTEM). Detailed TEM observations have revealed that no crystal structure change occurred under the protrusions during the repeated line-scratches; however, high density dislocations were found within the semicycle-shaped zone with a slightly dark contrast below protrusions (Figure 6a). This indicates that the lattice has been distorted and dislocations have been formed during the friction-induced fabrication. Selected-area electron diffraction (SAED) patterns from the original unaffected area (Figure 6b) and the deformed area (Figure 6c) showed the same  zone SiO_2 _pattern with the  plane parallel to the surface, as shown in Figure 6b. This indicates that the deformed area has the same single-crystal structure as that of original quartz. Unlike the friction-induced hillocks on silicon [19,20], no amorphous layer was observed under the friction-induced hillocks on quartz. The lattice distortion induced by the mechanical interaction may have played an important role in the formation of the protrusive nanostructures on quartz surfaces. However, the detail generation mechanism of the protrusion of material should be further investigated.

### Critical contact stress for the fabrication of friction-induced hillock

**Figure 7:**
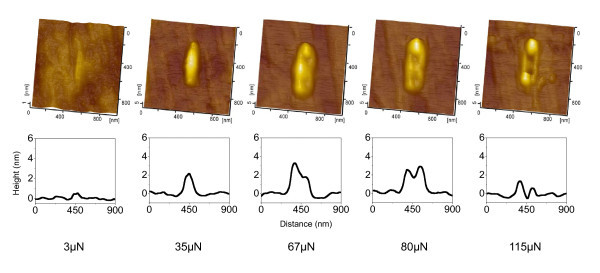
**Critical contact stress for the fabrication of friction-induced hillock**. AFM images (top) and cross-sectional profiles (bottom) of the friction-induced nanolines on quartz surface created by 30 scratching cycles at a displacement amplitude *D *of 200 nm.

It is well-known that quartz is essentially a stable oxide and hence no further oxidation can occur to it. As a result, the mechanical interaction should be responsible for the generation of hillocks on quartz. Under a high load, the scratching on material surface can lead to the generation of grooves [15,20]. Therefore, for a given AFM tip, it is important to select a suitable normal load for the fabrication of the protuberant nanostructures on quartz surface. As shown in Figure 7, under the conditions of tip radius *R *= 300 nm, *N *= 30 and *D *= 200 nm, the transition from the generation of the hillock to groove was observed with the increase in normal load *F*_n_. As *F*_n _increased from 3 to 35 μN, the hillocks kept growing from 0.4 to 2.5 nm. When *F*_n _reached to 67 μN, the peak of the structures began to collapse, but the formation of protrusive structures still dominated the scratching. When *F*_n _further increased to 80 μN, a slight groove was observed on the top of the hillock. Finally, when *F*_n _attained 115 μN, an evident groove was produced on the surface of quartz. Therefore, under the given conditions, 3 to 67 μN is the effective load range for the creation of protrusive structures on a quartz surface.

In fact, fabrication of those protrusive nanostructures depends strongly on both the applied normal load and the tip radius, namely the contact pressure between AFM tip and sample determines the ultimate appearance of hillocks in scratching [20,23,24]. In the present experiment, the Hertzian contact pressure *P*_c _can be estimated by [24,25]:(1)

With the equivalent elastic module *E *= 110.3 GPa [24,26-28], the tip radius *R *= 300 nm and normal load *F*_n _= 3 to 67 μN, the contact pressure *P*_c _for the fabrication of protrusive nanostructures on quartz is calculated to be 4.3 to 12.1 GPa.

On the other hand, the critical contact pressure *P*_y _for the initial yield of quartz can be estimated in the following. Since the Poisson ratio of quartz is 0.08 [27,28], the principal shear stress *τ_c _*was calculated by [25]:(2)

According to the Tresca yield criterion:(3)

the critical contact pressure *P*_y _can be calculated by:(4)

Since the yield stress *σ*_y _of quartz is 8.4 GPa [29], the critical contact pressure *P*_y _can then be estimated from Equation 4 as 11.7 GPa.

Therefore, it can be concluded that the contact pressure *P*_c _ranged from 0.4*P*_y _to *P*_y _is a feasible standard for friction-induced fabrication on a quartz surface under the given conditions. It reveals that the protrusive deformation is the initial surface damage during the nanowear on quartz. However, the surface damage of materials during nanowear process was not only depended on contact pressure *P*_c_, but also on the number of nanowear cycles *N*, displacement amplitude *D*, tribochemistry and other frictional factors [19,20,23,30]. When the experimental conditions change, the critical *P*_c _of the formation of hillocks may vary. For example, because of the serious tribochemical reaction during the nanowear of Si/SiO_2 _pairs in air, the scratches appeared on the silicon surface while the contact pressure was only 0.13*P*_y _under the experimental conditions of *N *= 200 and *D *= 12.5 nm [30].

### Selective etching of nanolines on quartz

**Figure 8:**
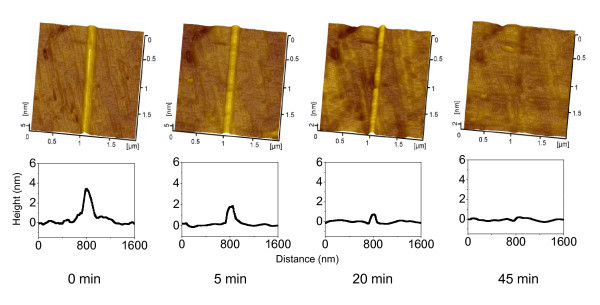
**Selective etching of the nanolines in KOH solution**. AFM images (top) and cross-sectional profiles of nanolines on quartz after etching in 20% KOH solution for various periods.

**Figure 9:**
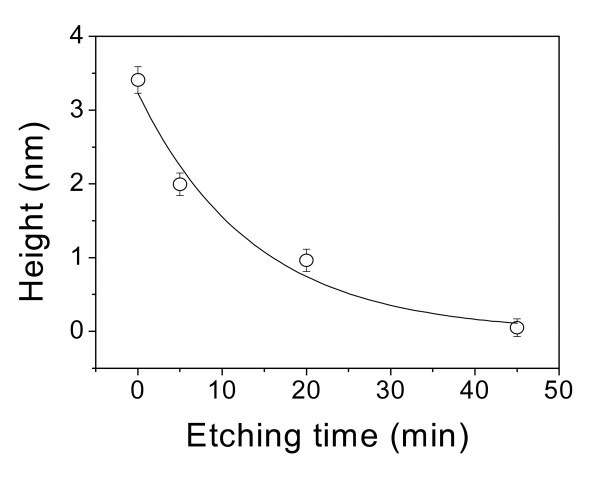
**The height of nanolines plotted as the function of etching time**.

In order to investigate the chemical activity of the friction-induced nanostructures on quartz surface, a quartz wafer with nanolines of 3.5 nm in height was dipped in 20 wt% KOH solution at 20 ± 3°C. The AFM images in Figure 8 show the topography and cross-sectional profiles of nanolines on quartz after etching in the 20% KOH solution for various periods. Figure 9 reveals the height of the friction-induced nanolines on quartz plotted as a function of the etching time in the 20% KOH solution. With the increase in the etching time, the height of nanolines decreased quickly in the first 5 min. Finally, the nanolines were dissolved after etching in the KOH solution for 45 min. The result implies that the friction-induced nanostructures have higher chemical activity than quartz substrate in KOH solution. It can be speculated that the selective etching of the nanolines is related to the stress-induced such defects as dislocations in the microstructures. According to the distribution of the contact stress, the hillock region has been undergone higher stress and has higher density of distortion than other dislocated area [24]. The chemical reaction of the hillocks may be enhanced by these dislocations, and the hillock regions are more readily dissolved in the KOH solution than other area [31]. As a result, the hillocks can be selectively erased in KOH solution after etching for an appropriate period. This character of friction-induced nanostructures may provide a potential rewriting technology in nanoscale: friction-induced fabrication is regarded as 'writing' while selective etching is 'erasing'.

In summary, the friction-induced method provides a manoeuvrable and direct way for fabricating protuberant nanostructures on quartz with high precision. Combining with the selective etching behaviour of the nanostructures, it is possible to develop a new nanofabrication technique on quartz.

## Conclusions

A novel friction-induced nanofabrication method has been developed to produce protrusive nanostructures on nonconductive quartz material. Under the given conditions, the height of the nanolines on quartz surface increases sharply and then tends to stabilize with the increase of the normal load or the number of scratching cycles. Since the nanolines generated in atmosphere and vacuum were almost same under the same loading conditions, the creation of friction-induced nanostructures on quartz may be mainly attributed to the mechanical interaction. Based on the TEM observation, the lattice distortion and formation of dislocations induced by the mechanical interaction was suggested to be the main generation mechanism of the protrusive nanostructures on quartz surfaces. The detailed analysis based on the experimental results and stress calculation reveals that, during scratching, a contact pressure ranged from 0.4*P*_y _to *P*_y _(*P*_y _is the critical yield pressure of quartz) is apt to produce protuberant nanostructures under the given experimental conditions. Finally, the protrusive nanostructures can be selectively dissolved in 20 wt% KOH solution. Since the nanowords can be easily 'written' by friction-induced fabrication and 'erased' through selective etching, it may open up new opportunities for future nanofabrication on quartz.

## Abbreviations

AFM: atomic force microscope; FIB: focused ion beam; MEMS/NEMS: micro/nano electromechanical systems; RMS: root-mean-square; SAED: selected-area electron diffraction; STM: scanning tunnelling microscope; TEM: transmission electron microscope; XPS: X-ray photoelectron spectroscope; XRD: X-ray diffraction machine; XTEM: cross-sectional transmission electron microscope.

## Competing interests

The authors declare that they have no competing interests.

## Authors' contributions

CS and BY realized the fabrication experiments and acquired the original data in this article. XL and HD done the TEM observation and analyse. LQ and ZZ have made substantial contributions to conception and design for this article.
